# Understanding Privatisation of Mental Health Services: Insights From Karl Marx's *Capital*

**DOI:** 10.1111/jpm.70032

**Published:** 2025-09-16

**Authors:** Myles Balfe

**Affiliations:** ^1^ Dept. of Sociology UCC Cork Ireland

## 1 Introduction

Considerable attention is being focused on the role, nature and consequences of privatised, or capitalist, health care in many countries and the role of nurses, doctors and other healthcare professionals in it. This is also the case in relation to the privatisation of mental health services (Guardian 2022; Silver‐Greenberg and Thomas 2024). For instance in the UK, it was noted in the early 2020s that the NHS—the leading international example of universal healthcare provision—was spending billions on private health care for mental health patients (Campbell and Bawden 2022). Karl Marx's book *Capital* (1867) provides ideas that are worth considering by mental health nurses who are interested in understanding the nature of privatised health care. This essay provides an overview of Marx's key concepts in *Capital* and how they can be used to interpret and make sense of privatised health care, mental and otherwise, and its risks. The 21st century is a time of extreme economic inequality and wealth concentration across the world, where capital is becoming increasingly dominant and embedded (McKeown 2024; Varoufakis 2023). This is a century where the interests of capital and capitalists are often taken as the interests of the wider public, health systems and states, and are increasingly coming to colonise these institutions. It is a time of increasing healthcare commodification and threats to universalist health care. It is a time that, increasingly, has features of Marx's time. Capitalism is now the dominant socioeconomic system in the world, so it can be useful for mental health professionals to understand it and how it can potentially impact their practice and working conditions.

## 2 Structure

The structure of this article is as follows. The first section discusses private health care as a form of what Marx refers to as a commodity. The article then discusses surplus value, which is the profit that is extracted from each commodity. After this, the idea of primitive accumulation is referenced, which refers to capitalistic colonisation of previously public entities. This is followed by a section on the impacts of capitalism on patients and mental and other health professionals. Two further concepts from Capital vol. 1 are then discussed. One is the concept of circulation, which describes the constant transformation of capital into money and commodities, and the other is constant and variable capital. The article concludes with some lessons that Capital vol. 1 might offer us in the 21st century, inheritors of this dying capitalistic world, where surplus value extraction has eclipsed all other ideals.

## 3 Private Healthcare as a Commodity

Marx argues in *Capital* that capitalist systems are commodity producing systems. A commodity is anything that is sold to satisfy some human want or need. Private health insurance, private health care, are examples; so would any other mental health care or medical service that is sold for profit. Healthcare commodities can therefore be goods or services. Actors who produce healthcare commodities say that they produce them for what Marx calls their use values (i.e., as a good in themselves) but they really produce them for their exchange value (i.e., for profit, or surplus value). Once the healthcare commodity is exchanged it is converted into its money form, where it stays or where it is reconverted back into another commodity. Money and commodities are, for Marx, different forms of the same thing, and all commodities are ready at any moment to escape their bodily form and assume their money form (Marx 1867, 33). So every private mental health service, from the perspective of capital, Marx says, is ‘in faith and truth money’ (Marx 1867, 107). He noted that if a commodity, including a healthcare commodity, could speak, it would say ‘our use value may be a thing that interests men. It is no part of us…in the eyes of each other we are nothing but exchange value’ (Marx 1867, 52). This constant transformation of money into healthcare commodities and back again is described by Marx using the formula M‐C‐M, or Money‐Commodities‐Money. Circulation is an end in itself in this system, has ‘a life process of its own’ (Marx 1867, 108).

The individuals who control the production of mental healthcare commodities in privatised systems are called healthcare capitalists. They employ healthcare workers such as mental health nurses, psychiatrists and allied mental health and psy‐ professionals. Capitalists own the means of healthcare production, the tools, infrastructure and technology that are needed to produce health care. Healthcare workers in privatised or semi‐privatised health systems use the means of production to generate medical commodities for capitalists that are then sold to patients, to other capitalists or to the state to generate surplus value, or profit. Marx would say that healthcare capitalists should never be seen to produce use values as an end in themselves; ‘the real aims of a capitalist is the never‐ending process of profit‐making’ (Marx 1867, 107). Healthcare capitalists generally employ significant numbers of mental health workers. There can be an intermediate group between healthcare employees and capitalists, which Marx called ‘small masters’, who might own a small number of small healthcare institutions that sell mental health services or commodities and then begin to expand as a result of patient demand or an inability to the state to provide core services.

## 4 Surplus Value

Capitalism for Marx is a system of biological and mental exploitation. It depends upon, feeds upon, the life force of workers, including healthcare workers, their ‘muscles, nerves, bones, and brains’ (Marx 1867, 121) and also the life force of patients who must purchase the healthcare commodities that those healthcare workers produce. Marx said that a capitalist institution, such as a private healthcare company, has one single life impulse, and that is the need to create surplus value. To do this it will, if it is allowed, make mental health nurses and other healthcare workers work longer (what Marx calls increasing absolute surplus value) and more intensively (what he calls increasing relative surplus value). Capitalism's need to do this is fundamental; it has a ‘werewolf's hunger for surplus value’ (Marx 1867, 179). It will seek to regulate what Marx called the socially necessary labour time that goes into the production of each healthcare commodity. Socially necessary labour time describes that average time that the average health professional of average ability would need to provide a medical or nursing service. If this time can be shortened, then more healthcare services can be produced and more surplus value extracted from the nurse's work. Healthcare work can also be outsourced to workers whose socially necessary labour time is cheaper than that of nurses (Moth 2020). This can enable relatively more surplus value to be extracted from those workers and put into circulation by the entity that employs them.

## 5 Primitive Accumulation

Capital will seek, where it is permitted, to privatise public health services and systems. This is what Marx refers to as primitive accumulation, a term that he uses to describe the capitalist colonisation of formerly public resources. Health care is attractive to capital because it is an example of a high‐profit sector with the ability to continually generate new commodities and where demand is inelastic (Braverman 1998/1974). It is also a sector with a high turnover time, which means that there is often not much time between supplying a service (a commodity) and receiving money for it. Basically capital does not stay long in the commodity form/production stage in healthcare; it goes through metamorphosis quickly. Once primitive accumulation has begun, capital will begin to produce the privatised medical commodities that are needed for life, and then over time begin to charge more and more money for them. Healthcare capitalists are bound by the laws of competition, as real to them as the laws of evolution, and must be pitiless in their pursuit of surplus value; otherwise, they themselves will be devoured by more aggressive life forms.

Some health systems are universalist in nature, which means in Marx's terms that health care is provided basically for free at the point of access, or at least for a low cost (i.e., health care is basically a use value), and the system has not been subjected to primitive accumulation on a mass scale. Other systems are fully privatised, which means that they have been successfully subjected to primitive accumulation. Others are a mix between public and private health care, which means that they have been partly subjected to primitive accumulation.

The state has a crucial role in controlling or enabling primitive accumulation. Capitalists will tend to seek primitive accumulation and privatisation. However, the state can, in theory, restrict this process. In the absence of state intervention, capitalism tends towards uncontrolled exploitation of the public (Galbraith 1963). A feature of many states over the past 40 years, however, is that many political and healthcare elites have come to accept, either out of ideology or their own financial interests, the perspective of capitalist elites and the logic of capitalism within health systems (Moth 2022). Marx noted that ‘whenever the legislature attempts to regulate the differences between masters and workmen, its counsellors are always the masters’ (Marx 1867, 524). All of this is an interesting, and recent, historical reversal in many states. Capitalism as a system preceded the emergence of modern public healthcare systems so in some ways privatisation of public health care is a return to an older per‐modern way of doing health. It is also, more immediately, a turn away from the more universal health and welfare systems that were developed after World War 2. After World War 2, capitalist states sought to prevent a return to the chaotic socioeconomic conditions that led to the rise of fascism and communism in the 1920s and 1930s (Harvey 2005). They developed a form of controlled capitalism that Harvey refers to as embedded liberalism, whereby capitalism was constrained, to a degree at least, by the state. As part of this process, capitalist states focused on things like providing universal health care to their citizens in order to ensure internal and external political stability. In the latter part of the 20th century, however, this form of capitalism was replaced across the world by a more socially Darwinian form of capitalism called neoliberalism. This form of capitalism argued that all public goods, such as health care, should be opened up to capitalist logics and should be fully privatised. Costs and exploitation of the public would be kept in check, or so the theory went, by subsequent competition amongst capitalist entities. The role of the state in this system was to represent the interests of business and corporations, and defend privatisation, including healthcare privatisation, from attempts to resist it (Waitzkin 1978). Under this system it is natural, as natural as breathing, to channel money from the state to private interests, and it is always at least thinkable to take money away from public services such as health care. In effect, this 21st‐century system of supposed perfected competition often amounts to socialism for the rich and capitalism for everyone else.

Once it has begun, primitive accumulation can manifest itself in various ways in a previously universalist health system. Certain health services, formerly provided by the state, might now be fully provided by private institutions. Or the state might act not as a supplier but as a procurer of health services from capitalist institutions (Moth 2022). Universalist health services might find themselves fully or partially dismantled (Moth 2020). Staff who work within these public health systems might find themselves forced to take on the ideas of capitalist health care in their everyday practice (Moth 2020).

Even partial primitive accumulation can be detrimental to universalist health systems. It can divert money towards private corporations and away from the public system. Over time, this will allow those corporations to generate more and more surplus value, which will allow them to expand, often at the expense of the public system. As capitalist health care becomes more present, more and more ordinary people will encounter it and come to see it as natural. Capitalist healthcare institutions invariably market themselves as high quality, personalised and caring institutions, in contrast to the opaque, dehumanising and labyrinthine public systems, and ordinary people might come to accept this view of the health system as the truth. Marx would say, though, that behind the images of appreciative children and smiling doctors and nurses lies a thing with ‘no heart in its breast’ (Marx 1867, 195).

## 6 Patients and Professionals

Marx would say that private health care is good for capitalists but it is, at the least, potentially bad for other groups, including patients. For instance, let us return to the point that all commodities have socially necessary labour time built into them. If a health professional or worker in a privatised system took longer to treat a patient, for example, spent an extra 30 min to talk and listen to each one, they would increase the socially necessary labour time that is being given per patient, which would undermine the generation of surplus value. The capitalist institution that employs them would probably not be happy. But patients value these types of interaction with healthcare professionals almost above everything else. Patients who have such interactions will often say about their health professionals that ‘they don't just see me as a number’. What such patients are saying is that you see me as having a use value, not just an exchange value.

Or alternatively, if a public health system has been starved of resources, for example as a result of austerity, and subjected to primitive accumulation, patients may feel that they need to pay to access health professionals who will treat them as having a use value. This is one of the reasons in fact why patients in some countries pay for things like private health insurance. Paradoxically, they can decide to pay to try to escape capitalist rationalisation of health care in public systems. In some nation‐states in the 21st century only those with money can access excellent quality mental health services.

Capitalists can be interested in improving the mental health of their workers, such as mental healthcare nurses, but also the mental health of patients who are themselves workers. The reason for this is that poor mental health can undermine productivity, which can lead to less commodities being produced and therefore less surplus value being generated. Marx noted that in his time capitalists ordered workers to eat food that was high in phosphate of lime as that increased their productivity. Today, an equivalent would be organisations encouraging workers to look after their mental health and focus on their well‐being, while not challenging the systemic pressures that are undermining workers' mental health and well‐being in the first place.

A problem for workers and patients, which Marx straightforwardly noted that capitalists simply do not care about (Marx 1867, 403), is that if the ‘necessaries of life consists of various commodities’ (Marx 1867, 222) then over time, as costs of these commodities go up, people will often have to work faster and longer to be able to afford them. This will in turn generate significant psychosocial stress and suffering at individual and population levels, which in turn will generate more acute mental health issues of various kinds. While capitalist health care is often presented as offering increased choice and personalised care to patients, working to make enough money to access it ‘usurps the time necessary for the healthy maintenance of the body…[and perhaps ultimately] the labourer's life’ (Marx 1867, 178).

Marx would also say that this system is also suboptimal for the health of nurses and other healthcare workers. In pursuit of absolute and relative surplus value capitalist healthcare institutions can drive healthcare workers beyond their biological limits to exhaustion, sometimes physical and mental ruination, sometimes suicide. It can force them to see patients as commodities, there for the purpose of surplus value extraction, not as real people in need of help. This will create despair in professionals who are not fully colonised by capitalistic logics. You just have to read any nursing or medical journal to note the frequency of articles published on things like burnout and moral injury amongst healthcare workers of all types (Guille and Sen 2024). Marx felt that this was a system ‘of terrible magnitude against life’. It is notable that the only biological life forms that he saw as being able to readily coexist alongside humans in this system were viruses.

Ultimately, Marx felt that capitalism, if it was not regulated, reduced human beings, healthcare workers, and patients, to a state of half‐life. Appendages of systems that they often do not fully understand, an ‘instrumentum vocale’, nurses and allied health professionals and patients generate surplus value while their life force is, over the course of their existence, drained away from them. Physically crippled in body and mind, life for many is reduced to a daily grind, an exhausted, basic struggle for existence where they work more and more intensively to either produce healthcare commodities or work to get the money to access them.

## 7 Circulation

The surplus value that is generated from the sale of healthcare commodities, including mental healthcare commodities, is used for key two purposes. One is to pay shareholders and owners of healthcare companies. The other is to intensify and expand the means of production by reinvesting surplus value, and thereby allow more private healthcare commodities to be produced; or possibly to acquire new means of healthcare production. For example the surplus value that is generated by the sale of private health insurance may be used to purchase a private hospital, which is used to then used to produce privatised mental healthcare services that are sold for profit, etc. Over time, if unregulated, the cyclical repetition of this process will mean that healthcare capitalists will begin to acquire more and more of the means of healthcare production. One thing that is always worth making note of in health systems that are partially subjected to primitive accumulation is when previously independent healthcare facilities begin to be bought up and become parts of chains or conglomerates; this is a sign that surplus value has been generated elsewhere and has been reinvested to acquire more of the means of healthcare production. It can mark the beginnings or continuation of genuine capitalist expansion. Marx noted that in any sector, including mental health care, capitalist circulation will eventually pass from a circular to an expansionary spiral form. Marx described the surplus value generated by this process of circulation as the ‘beautiful fruit of the vicious circle’ (Marx 1867, 478).

## 8 Constant and Variable Capital

Marx felt that this system was genuinely revolutionary, constantly seeking to increase absolute and relative surplus value, constantly seeking to develop and intensify the means of production. He noted that capitalists are, as a group, extremely interested in science and technology. He noted that technology is the key tool of capitalism as it enables biological limits on production to be overcome. The means of production are split into constant capital (for instance the tools and infrastructure of mental health care) and variable capital (for instance psychiatric nurses and other individuals who make health care). Capitalists seek to revolutionise constant capital in order to reduce their dependence on variable capital (i.e., to reduce their need for nursing labour (Braverman 1998/1974)), or to extract more surplus value from it, for instance, by reducing the amount of socially necessary labour time that each healthcare commodity requires. So, for instance, it is likely that capitalists would be interested in using advanced technologies like artificial intelligence to supply private mental health services as it would reduce their need to employ variable capital, that is, mental health professionals. Where permitted, such technologies will likely be used to direct, control and monitor nurses in their workplace so that their work can be made more efficient (i.e., they can process more patients more quickly) and they can produce more surplus value. This is a core function of technology under capitalism (Braverman 1998/1974; McKeown 1995).

Mental health professionals who were outside the capitalist mode of production (either totally, because they worked in a universal health system, or maybe partially if for example they had previously been an independent worker who was absorbed by a chain or corporation) but who are then brought into it experience what Marx (1864) refers to as subsumption. That means that their work becomes subsumed by capitalism and they become alienated from their work (alienation means that they have no final control over their work, they are subjected to absolute and surplus value processes and the ultimate goal of their employment is to produce surplus value, even though they would say that their job is to look after patients). This is what Marx called formal subsumption (see also Moth 2022) and means that a previously independent health practitioner becomes subjected to compulsion by a capitalist entity. The health practitioner might see benefits in this, for instance they might feel that the capitalist entity can do things like bureaucratic work and allow the health worker to spend more times with patients. However, the relation of compulsion and the need to produce surplus value are now there. At a certain point if the capitalist institution becomes developed enough it can make healthcare workers experience what Marx called real subsumption. This refers to a genuine transformation in the nature of the health professional's work. For instance, the healthcare worker might shift from being someone who exercised clinical judgement to someone whose practice is directly overseen and directed by managerial and increasingly artificial intelligences. Marx would probably say that in many cases the artificial intelligence is more human than the manager. Real subsumption for many health professionals can mean an escalating loss of autonomy and control over their work. Health systems can also experience formal and real subsumption. A health system that has been totally privatised will become something that is qualitatively and quantitatively different from the entity that it was before privatisation.

Nurses and other healthcare workers are probably, on the whole, more protected against revolutions in constant capital than other workers in other sectors. That does not mean that they are immune from such revolutions, just that they are probably more protected from them. It is important to note that a key goal of 21st‐century capitalism is to make knowledge workers, including healthcare workers, experience real subsumption, in much the same way that industrial workers experienced it in the 20th century. However, nurses are still impacted by these revolutions even if, for the moment, they do not directly make nurses themselves redundant. For instance, if constant capital is revolutionised in an industry and work is automated, a certain proportion of the workforce, what Marx calls the human biological mass, will be discarded. These people then become a surplus population, maybe employed elsewhere for less money, maybe forgotten. If entirely discarded, it is possible that such surplus populations will sink down to the bottom of the human organic layer where they will likely develop various mental health problems that nurses will then have to deal with, such as despair, drug abuse, alcoholism, depression, suicide, domestic violence, and so on (Zeira 2022). Health inequalities in such groups are not a sign that capitalism is working ineffectively—they are a sign that it has worked. Capital no longer needs the discarded population. In fact, discarded populations are useful for capitalism. They serve as a warning to those still in work about what will happen to them if they do not work long enough or hard enough (though whether you work long or short, the revolutionary drive in capitalism means that, for many, your destiny will ultimately be the same). They also act as a reserve workforce that can suppress the value of labour power (Marx 1867, 288). This has the effect of making healthcare commodities more unaffordable for those still in work.

## 9 Lessons for Mental Health Professionals

What lessons should we draw in the 21st century from Marx's *Capital*? I think that is absolutely clear that he would support the strengthening of public health systems that provide universal mental health care (though the risks of the socialist mental healthcare systems that were generated in the wake of Marx's ideas are also clear and, like capitalist health care, need to be managed). Private health care he would view as being more problematic, to say the least. Even if you feel that you are producing private mental health care to help people (as a use value) Marx would say that you are really selling it (producing exchange value). If a patient could not pay, would you still treat them? And is that right? The other issue is that once health care is commodified the most common way to extract surplus value from it is to increase the price and decrease the service over time. If you scale this process out with compound interest eventually there will come a time when many patients, even those relatively well‐off, will be unable to afford such health care. This will probably happen in the medium term, not the long‐term. Marx notes that at a certain point the price of commodities, including mental healthcare commodities, will cease ‘altogether to express value’ (Marx 1867, 70). If the health system has been subjected to primitive accumulation by that point, privatised to a large extent, many patients, not just the unemployed or the socially forgotten, will find themselves in serious trouble.

Mental health nurses who work in highly privatised medical systems, or even semi‐privatised systems, must make sense of practising medicine under capitalism. Health care for many nurses is a vocation, practised for its own sake—which means that is a use value. Capitalist health care is produced for exchange value. These two things are in conflict; the increasing corporatisation of health care, for example, has been referred to as a ‘marriage made in hell’ (Hoffer 2024). This conflict might not immediately manifest if you are a private sector healthcare worker who is dealing with patients who can afford to pay, but it will, or at least it should, appear if they cannot. Nurses have noted that many healthcare professionals are either intentionally or unintentionally ignorant of, or simply indifferent towards, capitalist forces in health care (McKeown et al. 2017). This is not surprising. Capitalism is intentionally mind‐numbing (Varoufakis 2023). That there is no alternative to capitalist logic is a message that has been pushed for decades. Healthcare workers now come into a world where capitalism has already, for the moment at least, triumphed (Braverman 1998/1974). Marx felt that anyone who was upset by capitalism without seeking to do anything about it was engaging in a ‘very cheap sort of sentimentality’ (Marx 1867, 122).

However, while capitalism itself as a global system might not itself be controllable (it might not even be desirable to fully control it‐ to be honest, there are very different ideas about what to control capitalism even means), or there might be no genuine alternatives to it at this point in time, researchers have felt that it can be controlled in relation to health care (Moth and McKeown 2016). By control here, I mean regulate its presence in health systems so that the majority of care is provided to patients either free or at low cost at the point of access and whereby patients are able to access such care readily without substantial barriers such as long waiting lists or bureaucratic hurdles. Health care then would be basically provided to patients as a use value. I think that there are two fundamental reasons why this can be done. One is the fact that even if healthcare workers are subjected to subsumption, there will be part of at least some of them that became healthcare workers for vocational reasons. This part of them will be resistant to capitalist logic, even if this part is dormant within them. The other reason is that I think that across the world and across time the public have an understanding that health care is different from other commodities. Even in highly privatised systems, people frequently express outrage when healthcare corporations and insurance companies are perceived to be profiteering off of the sick and the vulnerable. Significant efforts sometimes have to be made by such corporations and by their client governments to suppress feelings of societal outrage towards privatised health care. Since professionals and patients are the two most important groups in health care (the ones supplying the service and the ones paying for it; everyone else is, from Marx's perspective, simply feeding on their surplus value), there will always be at least the possibility of change and a shift towards more universalist health care even in highly privatised systems.

So what can be done? Mental health and psychiatric nurses need to defend health institutions that provide universal care. This must be done consistently and assertively and involve ongoing messaging to the public and professionals about the benefits of universal and public healthcare. Health professionals must be prepared to struggle, in an ongoing way—this is a fight that will never be definitively won—to demand accessible public health care for all. They must see this as part of their core professional ethos. There is a simple and powerful message for the public here that health professionals can use in this practical and ideological struggle. You have a choice. You can receive excellent quality health care, mental or other, that is provided by skilled and compassionate nurses and doctors who are concerned about you, who do not care about how much money you have, which is free, or at least low cost, at the point of access. You will be guaranteed to be looked after when you become sick. Or you can pay for that health care, the cost will increase every year, the struggle to understand the complexity of the private system will be placed on you, and if you are a middle‐income or lower earner you will struggle to afford it when you are old or sick and when you most need it.

Healthcare professionals need to develop strong and resilient connections with one another that are based around shared commitments to public systems, systems that are insulated against the demands of capitalism. They need to ally with and support political movements that seek to control privatised health care. These connections are important as attempts to control, let alone reverse, primitive accumulation will likely, in some cases at least, generate retaliatory responses by privatised healthcare institutions and by those ideologically or practically committed to privatised health care. No one should be naive about what is going on here; there is a lot of money at stake. For their part, to the extent that they can, health professionals should base their practice around the concept of use value and not exchange value, which is easier said than done, but crucial to generating public support. States, for their part, need to support doctors and nurses by supplying them with well‐paid and secure jobs where they are valued and supported so that they do not feel that they need to go into privatised health care, or turn to privatised health care to manage things like increased patient demand or to reduce waiting lists. Capitalist logics that involve surreptitious privatisation, such as outsourcing, need to be publicly identified as they are happening; they should not be allowed to proceed secretively. If a healthcare company, such as a private hospital or private health insurance company, is marketing private health care, then nurses need to respond by highlighting the risks of the commodity that is being sold; in effect, health warnings need to be placed around privatised health care. Its claims need to be challenged and it should not be seen as the natural or the only way of providing health care. Regulatory capture can be controlled in various ways; for example, by legislating to prevent health policy makers or health professionals who work for the state from subsequently working for companies that work or invest in private health care, or vice versa.

Mental health nurses and other professionals need to clarify for themselves what they see as acceptable levels of privatisation within health systems; and where, and if, they think that privatisation should occur and what its nature should be (i.e., provide entire classes of services or support for particular services). Private healthcare services obviously can have benefits. However, as outlined here, private health care will always be governed by the need to generate surplus value. That is its primary concern. Health care is almost incidental to that. Health professionals should not drift into situations where they are surprised that half of the health care in a system is now controlled by private interests. At the point that creeping privatisation is embedded, then entire classes of professional practice will be subjected to real subsumption. In health systems that have already been subjected to high levels of primitive accumulation, health professionals need to clarify for themselves as a group the extent to which they want to actively resist and attempt to roll back that accumulation and how they would proceed in doing that.

Ultimately, achieving universal, genuinely inclusive and progressive health care in a capitalist system will only be achieved through ongoing collective action by health professionals, patients and key stakeholders such as politicians who support (and are made to continue to support) the idea of universal health care (McKeown 2009). Collective action is essential as capitalist health entities are likely themselves already engaged in collective action in support of privatisation, through lobbying and representative groups, think tanks, connections with the media and universities and so on. An individual health professional will not on their own be able to resist the weight of money and influence that is presented by those entities. It is only as a group, based around shared ideals and a commitment to universal health care, to a commitment to providing all of the public, not just those with money and connections, with the gift of genuine care (Kuhling and Keohane 2012) that healthcare professionals will be able to resist privatisation efforts and bring into being a different world, a fairer, better world, a world that is not based on the economic exploitation and commodification of the fearful, the sick and the vulnerable.

Perhaps though as a very first step Marx would say that Mental Health Nurses in capitalist systems who are concerned about the commodification of health care in the 21st century need to realise the nature of the system that they are in; and that this system may be hostile to them, to their patients, to anyone who seeks to disrupt the generation of surplus value. Health care, including mental health care, privatisation is expanding in many nation states in the 21st century. As an individual Nurse or healthcare professional, you might not necessarily be able to do anything immediately about this system. However Marx would say that maybe as a first step, you should be straight with yourself about the nature of the system that you are in, maybe supporting, and what it is doing to you, your patients; and maybe, ultimately, if it ever comes time for them to seek mental health care, what it will do to your children.

The first step therefore is awareness. As is noted on Marx's gravestone, though, understanding the world is only the beginning.

The point of understanding the world is to change it.

## Conflicts of Interest

The author declares no conflicts of interest.

## Data Availability

Data sharing not applicable to this article as no datasets were generated or analysed during the current study.
